# Complete Genome Sequence of the Type Strain of the Acetogenic Bacterium *Moorella thermoacetica* DSM 521^T^

**DOI:** 10.1128/genomeA.01159-15

**Published:** 2015-10-08

**Authors:** Anja Poehlein, Frank R. Bengelsdorf, Carola Esser, Bettina Schiel-Bengelsdorf, Rolf Daniel, Peter Dürre

**Affiliations:** a Genomic and Applied Microbiology & Göttingen Genomics Laboratory, Georg-August University, Göttingen, Germany; b Institut für Mikrobiologie und Biotechnologie, Universität Ulm, Ulm, Germany

## Abstract

Here we report the closed genome sequence of the type strain *Moorella thermoacetica* DSM 521^T^, an acetogenic bacterium, which is able to grow autotrophically on H_2_ + CO_2_ and/or CO, using the Wood-Ljungdahl pathway. The genome consists of a circular chromosome (2.53 Mb).

## GENOME ANNOUNCEMENT

Homoacetogenic organisms are able to grow autotrophically on H_2_ + CO_2_ by using the Wood-Ljungdahl pathway. *Moorella thermoacetica* DSM 521^T^, formerly *Clostridium thermoaceticum*, which was isolated from horse feces in 1942 (1), was used as a model acetogen for elucidating the Wood-Ljungdahl pathway. Mainly two strains, *M. thermoacetica* DSM 521^T^ and *M. thermoacetica* strain ATCC 39073, were used. For example, strain *M. thermoacetica* DSM 521^T^ was used to study the metabolism of carbohydrates (2), features of acetate kinase (3), the presence of cytochrome and menaquinone (4), and use of CO as electron donor (5). The biochemistry for reduction of H_2_ + CO_2_ and**/**or CO using the Wood-Ljungdahl pathway is described in detail in a number of reviews (6, 7). In some studies, cells or chromosomal DNA of *M. thermoacetica* DSM 521^T^ were used, but the genome sequence of the non-type strain *M. thermoacetica* ATCC 39073 (8) was cited (for example see references 9 and 10). Therefore, we decided to sequence and publish the genome of the type strain *M. thermoacetica* DSM 521^T^.

A MasterPure complete DNA purification kit (Epicentre, Madison, WI, USA) was used to isolate chromosomal DNA of *M. thermoacetica* DSM 521^T^. Isolated DNA was used to generate Illumina shotgun sequencing libraries. Sequencing was performed with a MiSeq system using a MiSeq reagent kit v3 (600 cycles) as recommended by the manufacturer (Illumina, San Diego, CA, USA). Sequencing resulted in 4,553,522 paired-end reads (2 × 300 bp) that were trimmed using Trimmomatic 0.32 (11). *De novo* assembly performed with the SPAdes genome assembler software version 3.5.0 (12) yielded in 40 contigs (>500 bp) and an average coverage of 382-fold. For scaffolding, we used the Move Contigs tool of the Mauve genome alignment software (13) and the genome of *M. thermoacetica* ATCC 39073 (CP000232) as reference. Remaining gaps were closed by PCR-based techniques, primer walking, and Sanger sequencing of products using BigDye 3.0 chemistry and an ABI3730XL capillary sequencer (Applied Biosystems, Life Technologies GmbH, Darmstadt, Germany). For this purpose, the Gap4 (v4.11) software of the Staden package (14) was employed.

The genome of *M. thermoacetica* DSM 521^T^ consists of a circular 2.53-Mb chromosome with an overall G+C content of 55.95%. Automatic gene prediction was performed by using Prodigal software tool (15). Genes coding for rRNA and tRNA were identified using RNAmmer (16) and tRNAscan (17), respectively. An Integrated Microbial Genomes–Expert Review (IMG-ER) system (18) was used for automatic annotation, which was subsequently manually curated by using the Swiss-Prot, TrEMBL, and InterPro databases (19). The genome harbored 3 rRNA genes, 52 tRNA genes, and 2,553 protein-encoding genes. Genes encoding proteins of the carbonyl branch of the Wood-Ljungdahl pathway showed the same arrangement as in *Moorella thermoacetica* ATCC 39073. Comparison revealed that the genome of strain DSM 521^T^ (2,527,564 bp) is smaller than that of ATCC 39073 (2,628,784 bp) and that of the recently published *Moorella thermoacetica* DSM 2955^T^ (2,623,349 bp) (20). We detected 992 single nucleotide polymorphisms in the final genome compared to *M. thermoacetica* ATCC 39073.

### Nucleotide sequence accession number.

This complete genome project has been deposited at DDBJ/EMBL/GenBank under the accession number CP012369.
